# *Oreocharis scutifolia* (Gesneriaceae), a Peltate‐Leaved New Species From the Dry–Hot Valley of the Jinsha River Basin, Yunnan, China

**DOI:** 10.1002/ece3.70442

**Published:** 2024-10-24

**Authors:** Zhi Xie, Na‐Na Peng, Miao Zhang, Guo‐En Ding, Fang Wen, Hang‐Hui Kong

**Affiliations:** ^1^ Key Laboratory of National Forestry and Grassland Administration on Plant Conservation and Utilization in Southern China, South China Botanical Garden Chinese Academy of Sciences Guangzhou China; ^2^ South China National Botanical Garden Guangzhou China; ^3^ University of Chinese Academy of Sciences Beijing China; ^4^ Department of Biological Science College of Life Sciences, Sichuan Normal University Chengdu China; ^5^ Guangxi Key Laboratory of Plant Conservation and Restoration Ecology in Karst Terrain Guangxi Institute of Botany, CAS Guilin China; ^6^ National Gesneriaceae Germplasm Resources Bank of GXIB, Gesneriad Committee of China Wild Plant Conservation Association Gesneriad Conservation Centre of China (GCCC), Guilin Botanical Garden, CAS Guilin China

**Keywords:** new taxon, *Oreocharis scutifolia*, peltate leaves, taxonomy, the dry–hot valley

## Abstract

A peltate‐leaved new species, *Oreocharis scutifolia* Z. Xie & H. H. Kong, endemic to the Dry–Hot Valley of the Jinsha River Basin, Yunnan, China, is described and illustrated here. It is similar to *O. cordatula* (Craib) Pellegr. and *O. aurantiaca* Baill. in floral characters but differs in its peltate leaf blades, which are unique in the genus (and only occur in one population of *O. henryana* Oliv., but its flowers are smaller, campanulate, and deep purple). Molecular phylogenetic analysis based on transcriptome data confirmed its systematic position to be sister group with *O. henryana*, *O. cordatula*, *O. minor* Pellegr., and *O. aurantiaca* Baill. (LPP = 1), and well apart from the remaining members of the genus. The new species *O. scutifolia* is assessed as “Critically Endangered” following the IUCN categories and criteria, due to its small and single population, thus making it face serious threats from human disturbance, invasive plants, shrinking habitat, and decreasing habitat quality.

## Graphical abstract

A peltate‐leaved new species, *Oreocharis scutifolia* Z. Xie & H. H. Kong, endemic to the Dry–Hot Valley of the Jinsha River Basin, Yunnan, China, is described and illustrated here. It is similar to *O. cordatula* (Craib) Pellegr. and *O. aurantiaca* Baill. in floral characters but differs in its peltate leaf blades. Molecular phylogenetic analysis based on transcriptome data confirmed its systematic position to be sister group with *O. henryana*, *O. cordatula*, *O. minor* Pellegr., and *O. aurantiaca* Baill. (LPP = 1), and well apart from the remaining members of the genus. The new species *O. scutifolia* is assessed as “Critically Endangered” following the IUCN categories and criteria.

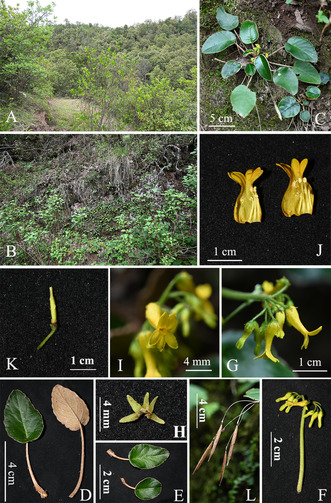

## 1 Introduction

**FIGURE 1:**
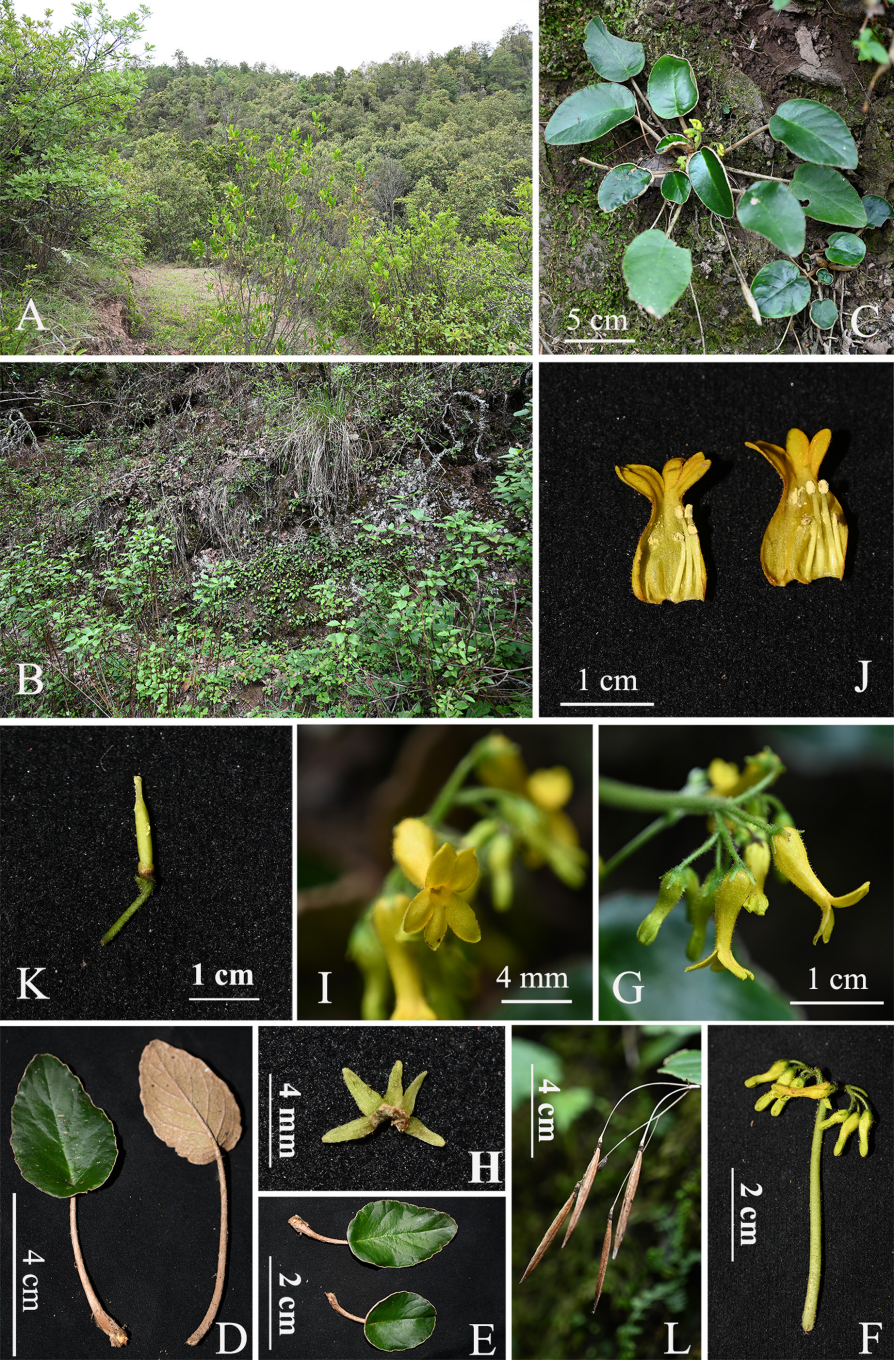
*Oreocharis scutifolia* Z. Xie & H. H. Kong sp. nov. (A) habitat, the *Quercus franchetii* Forests; (B) population, invaded by *Ageratina adenophora*; (C) habit; (D) mature leaves: Adaxially blade (left) and abaxially blade (right); (E) immature leaves; (F) Cyme; (G) left side view of the flowers; (H) Calyx; (I) front view of flower; (J) opening corolla, showing stamens and staminode; (K) pistil and disk; (L) Dehiscent capsules. Photographs by Zhi Xie.

*Oreocharis* Benth. (Gesneriaceae) is a species‐rich genus distributed from Eastern Himalaya to China, Indo‐China, and Japan (Möller et al. 2011; Kong et al. 2022). There are a total of 155 species and 15 varieties of *Oreocharis*, with 144 of these species, and all varieties are found in China (according to the Gesneriaceae Resource Centre, https://padme.rbge.org.uk/grc/, last accessed on June 10, 2024). Almost all currently known species of *Oreocharis* grow on shady and wet rocks or in stony soils (Kong et al. 2022). When the environment becomes dry, these plants do not grow well, or even dry up, and cannot bloom normally. An interesting phenomenon observed in *Oreocharis*, as well as in other Gesnerids, such as *Boea hygrometrica* (Bunge) R. Br. (Xiao et al. 2015) and *Corallodiscus lanuginosus* (Wall. ex A. DC.) B. L. Burtt (as noted in our field investigation), is their ability to resurrection from dehydration, extreme brittleness, and even breakage when supplemented with water. Therefore, these plants are called “resurrection plants” (Xiao et al. 2015). In July 2023, we unexpectedly discovered an unknown and unique species of *Oreocharis* (Gesneriaceae) during a field investigation in the Dry–Hot Valley of the Jinsha River Basin, Dayao county, Yunnan, China. These plants grow on soil slopes or damp rocks. The habitat is dominantly occupied by *Quercus franchetii* Skan (Fagaceae) and is accompanied by species such as *Petrocosmea forrestii* Craib (Gesneriaceae), *Deyeuxia mazzettii* Veldkamp (Poaceae), *Arisaema erubescens* (Wall.) Schott (Araceae), and *Thalictrum* sp. (Ranunculaceae) (Figure 1A,B).

**FIGURE 2:**
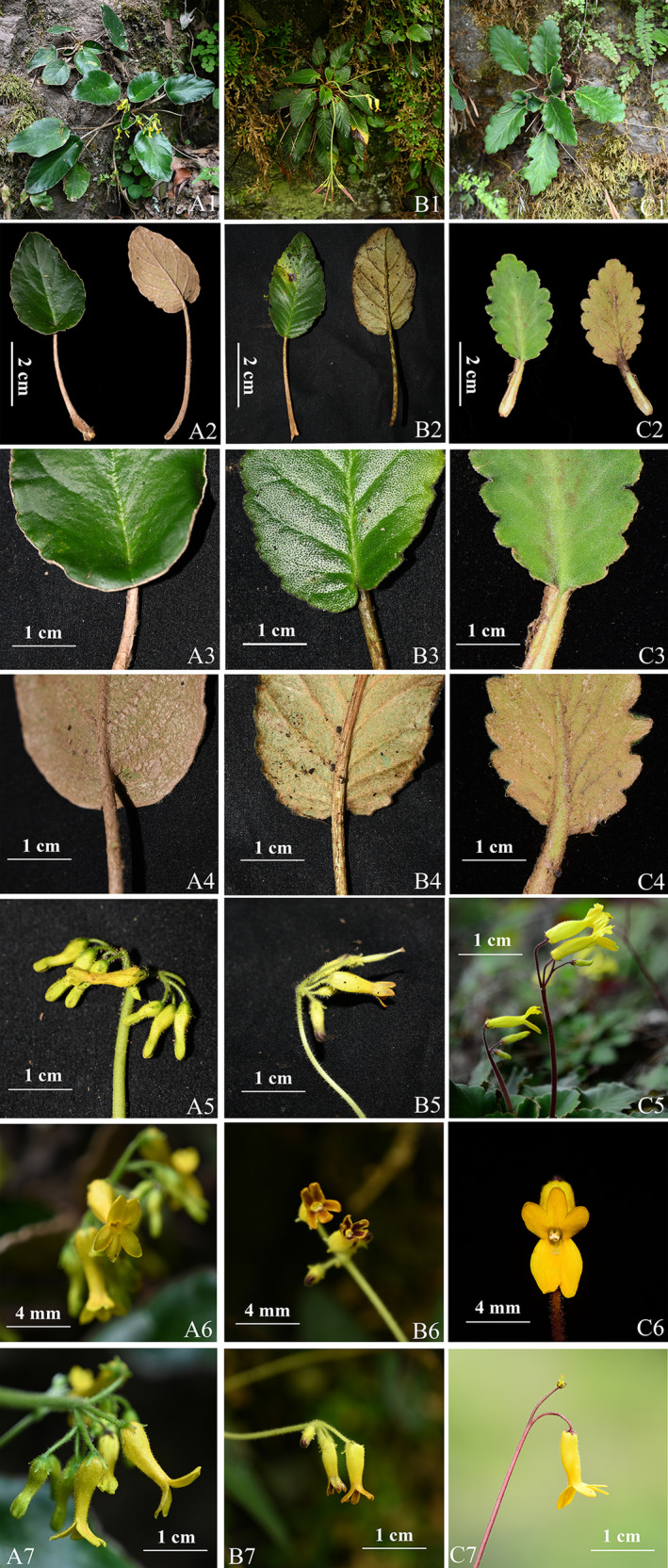
Comparison of *Oreocharis scutifolia* (A1–A7), *O. cordatula* (B1–B7) and *O. aurantiaca* (C1–C7). A1, B1, and C1, habit. A2, B2, and C2, leaves: adaxially blade (left) and abaxially blade (right). A3, B3, and C3, leaf base: adaxially blade. A4, B4, and C4, leaf base: abaxially blade. A5, B5, and C5, Cymes. A6, B6, and C6, front view of flowers. A7, B7, and C7, left side view of the flowers. C3, C4, C5, and C7 were taken by Xin‐Xin Zhu; C6 was taken by Hang‐Hui Kong; and other photographs were taken by Zhi Xie.

**FIGURE 3:**
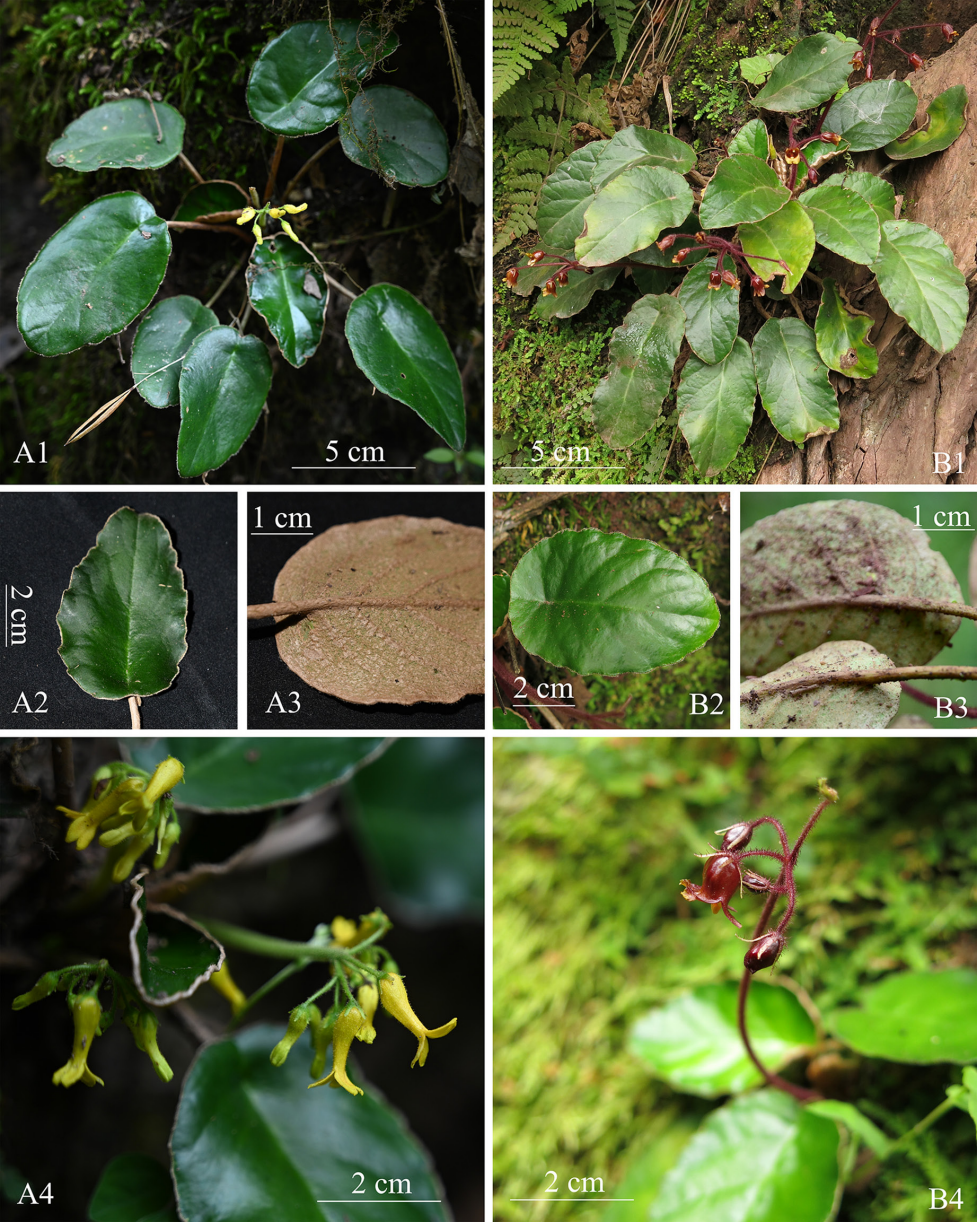
Comparison of *Oreocharis scutifolia* (A1–A4) and *O. henryana* with peltate leaves (B1–B4). A1 and B1, habit. A2 and B2, adaxially leaf blade. A3 and B3, abaxially leaf blade. A4 and B4, Cymes and flowers. A1–A4 were taken by Zhi Xie, and B1–B4 were taken by Li‐Hua Yang.

**FIGURE 4:**
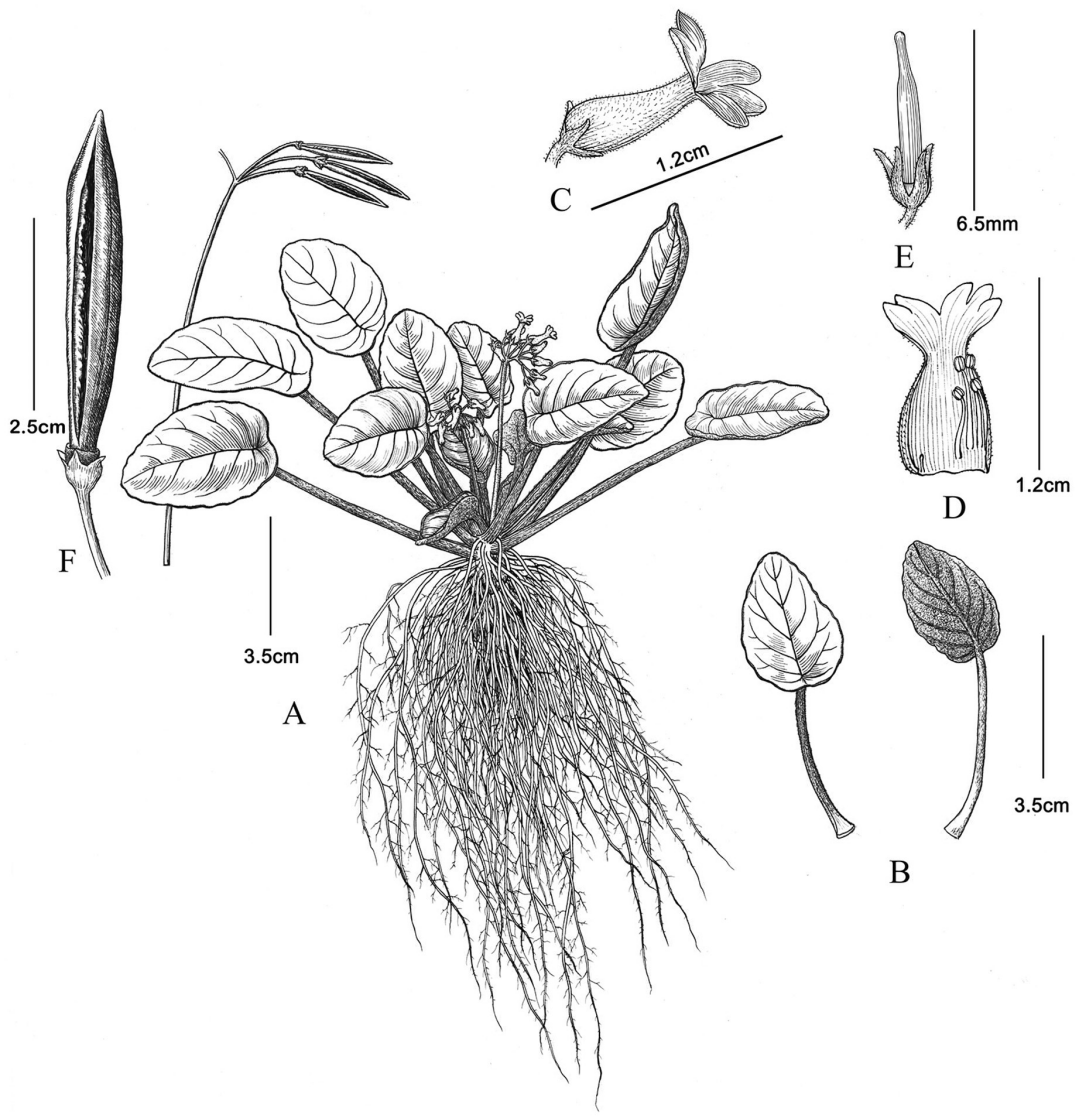
*Oreocharis scutifolia*. (A) plants with flowers and dehiscent capsules; (B) adaxial leaf (left) and abaxial leaf (right); (C) flower; (D) opening corolla showing stamens and staminode; (E) pistil with sepals; (F) dehiscent capsule. Drawn by Yun‐Xiao Liu.

The newly collected plants exhibit distinctive characteristics, including rosette habit, basal leaves, axillary cymose inflorescence, slenderly cylindric corolla and 2‐lipped limbs, 4 stamens and 1 staminode, and annular disk. These traits align with the morphological description of *Oreocharis* (Wang et al. 1990, 1998; Möller et al. 2011). They also share some similar morphological characteristics with *O. cordatula* (Craib) Pellegr. and *O. aurantiaca* Baill. but can be distinguished by their unique peltate leaves. This characteristic sets it apart from the aforementioned two species (Figure 2) and any other *Oreocharis* species. Additionally, during our research, we encountered one population of *O. henryana* Oliv. with peltate leaves on wet rocks in Miyi county, Sichuan, China (located on the roadside of the way from Wantan Hydropower Station to Malong Township, at an elevation of 1270 m, 13 September, 2017, collected by H. H. Kong, L. H. Yang, and B. F. Zhou with accession number SCMY04 at IBSC). Its purple to deep purple flowers can also distinguish it from our newly collected plants (Figures 3 and 4). Detailed literature reviews and morphological comparisons with herbarium specimens confirmed that this new collection is a unique and previously undescribed species. Phylogenetic analysis, exclusively utilizing single‐copy nuclear genes extracted from transcriptomes, has confirmed its systematic position of the genus *Oreocharis* (Figure 1).

## 2 Material and Methods

### 2.1 Morphological Description and Comparison

Morphological data for the new species were collected from field observations of living plants in their natural habitat. We also measured the morphologically similar species, including *O. cordatula* and *O. aurantiaca*, which have leathery leaves with yellow flowers, both in the field and from the specimens at PE, IBSC, and KUN. Morphological measurements were taken using a vernier caliper from Yantai Greenery Tools Co., Ltd. (Yantai, China) to document specific characters relevant to species differentiation. The terminology used to describe morphological characters followed the guidelines proposed by Harris and Harris (1994) and the Flora of China (Wang et al. 1990, 1998). Voucher specimens for our study, including the type materials, were deposited in the herbarium of the South China Botanical Garden, Chinese Academy of Sciences (IBSC), Guangzhou, China. In our examination of *Oreocharis* (as previously defined prior 2011) specimens, we comprehensively reviewed collections from various herbaria. Our evaluation encompassed both physical specimens (including those at IBSC, PE, CDBI, IBK, and KUN) and digital images accessible via the National Plant Specimen Resource Center (www.cvh.ac.cn/index.php, including herbarium of SM, NAS, GZTM, CSH, HGAS, and WGSBG) and the JSTOR Global Plants web portal (https://plants.jstor.org/, including herbarium of K, E, P, MPU, and UC).

### 2.2 Molecular Analyses

In this study, 128 samples representing 106 *Oreocharis* species (Table S1) were used for phylogenetic analyses, including the new species, *O. aurantiaca*, *O. cordatula*, and *O. henryana* Oliv., along with six species from other genera of Gesneriaceae (*Cyrtandra hawaiensis* C.B.Clarke, *Petrocodon dealbatus* Hance, *Didymocarpus cortusifolius* [Hance] W.T. Wang, *Anna mollifolia* (W.T. Wang) W.T. Wang et K.Y. Pan, *Aeschynanthus moningeriae* [Merr.] Chun and *Aeschynanthus buxifolius* Hemsl.) as outgroups based on previous molecular phylogenetic analyses (Möller et al. 2011; Kong et al. 2022). Six samples were newly collected in this study, including the new species (collected from the type locality), *O. cordatula* (collected from Jiulong county, Sichuan, China), three populations of *O. henryana* with normal leaves (all collected from Sichuan, China), and one population of *O. henryana* with peltate leaves (collected from Miyi county, Sichuan, China). For each population, we collected one or more whole living plants ensuring their roots and lower portions were gently enclosed with damp paper towels or, if available, with damp moss (Kong et al. 2022). The collection date, location, habitat, altitude, latitude, and longitude were meticulously recorded at the same time. Subsequently, we transported these living plants to the greenhouse at South China Botanical Garden, Chinese Academy of Sciences. Once the plants recovered, we collected fresh young leaves, which were then rapidly put into liquid nitrogen, and stored in a − 80°C freezer.

We submitted the frozen samples to Novogene Technology Co., Ltd. for transcriptome sequencing. We employed fastp (Chen 2023) to eliminate low‐quality reads with parameter ‐*g* ‐*q* 5 ‐*u* 50 ‐*n* 15 ‐*l* 150 ‐*overlap_diff_limit* 1 ‐*overlap_diff_percent_limit* 10. We then referenced a previously published dataset, which included 574 orthologous putative single‐copy genes and covered 106 *Oreocharis* species, and extracted the relevant orthologous genes by GeneMiner (Xie et al. 2024) to obtain a new dataset (see support file on Figshare, 10.6084/m9.figshare.26927632.v1), which including 50% missing taxa and 25.1% missing data. We aligned the orthologous genes using MAFFT 7.525 (Nakamura et al. 2018) with default settings, and removed ambiguous regions with Trimal 1.4.rev15 (with the argument**–**automated1) (Capella‐Gutiérrez, Silla‐Martínez, and Gabaldón 2009). Phylogenetic inference was first performed with IQ‐TREE 2.0.4 (Minh et al. 2020) based on local posterior probability (LPP) algorithm for each orthologous gene separately. The resulting gene tree files were then input into ASTRAL‐III 5.7.8 (Zhang et al. 2018) to reconstruct the species tree.

## 3 Results

### 3.1 Morphology

**FIGURE 5:**
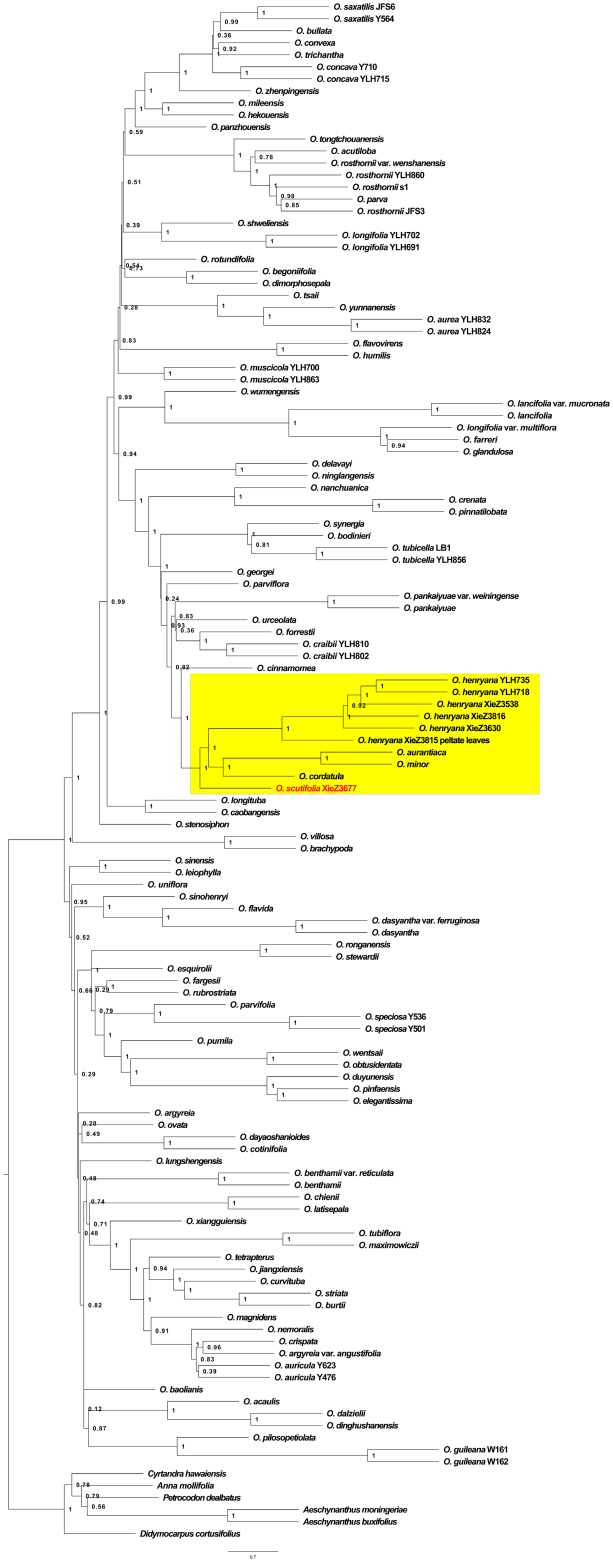
Phylogeny of *Oreocharis* based on the transcriptome dataset of 574 single‐copy genes. The new species is shown in red, with the branch on which it was placed marked in yellow. The species tree was reconstructed by ASTRAL‐III 5.7.8. Support for branches was evaluated with local posterior probability (LPP).

A morphological comparison of the new species with known *Oreocharis* species revealed a remarkable resemblance to *O. cordatula* and *O. aurantiaca* sharing common characters, that is, simultaneously possessing the leathery leaf blades and yellow flowers (Figures 1 and 5). However, it can be distinguished by its distinctive peltate, ovate to oblong leaves, the petiole adnate to the margin of leaves approximately 0.5–1 cm from base, margin shallowly undulate to almost entire, adaxially smooth and hairless, peduncles 3–7 cm long, and corolla 1.1–1.3 cm long. A comprehensive comparison of the morphological characteristics of similar species is presented in Table 1.

**TABLE 1:** Morphological comparison among *O.scutifolia*, *O.cordatula*, *O.aurantiaca*, *O.minor,* and *O.henryana*.

Characters	*O. scutifolia*	*O. cordatula*	*O. aurantiaca*	*O. minor*	*O. henryana*
Habitat	On rocks or soils under the semi‐humid evergreen broad‐leaved forests	On rocks or soils under the humid evergreen broad‐leaved forests	On rocks under the dark and damp forests	Shady rocks on cliffs in valleys	On rocks or soils under the humid evergreen broad‐leaved forests
Leaf blade					
Shape	Peltate, oblong to ovate, even rounded in the inner leaf	Ovate‐lanceolate to narrowly ovate	Rhombic‐ovate to narrowly elliptic or lanceolate	Rhombic‐ovate to elliptic or lanceolate	Narrowly oblong to lanceolate, rarely peltate
Adaxial surface	Smooth and hairless	Densely appressed pubescent	Densely appressed pubescent	Glabrous	Pubescent to densely pubescent
Abaxial surface	Densely brown manicate lanose	Densely pale brown wooly	Sparsely to densely pale brown wooly	Densely pale brown wooly	Densely light brown pannose
Margin	Shallowly undulate to nearly entire	Coarsely crenate to coarsely serrate	Coarsely crenate to coarsely dentate or serrate	Coarsely crenate to coarsely dentate or serrate	Irregularly crenate to serrate or dentate
No. of lateral veins	5–6 pairs	5 or 6 pairs	3–7 pairs	4–6 pairs	5–7 pairs
Peduncle indumentum	Densely covered with white glandular pubescence	Translucent to purple‐red glandular pubescent	Translucent to purple‐red glandular pubescent, glabrescent	Translucent to purple‐red glandular pubescent	Glandular pubescent to villous
Bract	Bract usually not present	2, deciduous, tiny, usually absent ovate to linear, pubescent	2, deciduous, tiny, usually absent, ovate to linear, pubescent	2, deciduous, ovate to linear, pubescent	2, linear to subulate, often deciduous, villous
Corolla limp					
Color and size	Yellow, 1.1–1.3 cm	Deep orange to yellow, 1.9–2.4 cm	Deep orange to orange, 1.6–2.5 cm	Yellow, 1.3–1.6 cm	Purple to deep purple, 7–11 mm
Adaxial lip lobes size	1.4–1.6 × 1.5–1.8 mm	3–4 × 3–5 mm	2.5–4 × 2–3 mm	3–4 × 2–3 mm	All lobes 2–4 × 1.5–3 mm
Abaxial lip lobes size	2.3–2.9 × 1.3–1.5 mm	6–7 × ca. 2 mm	7–8 × 1–2 mm	3–4.5 × 1.5–2 mm	
Stamens					
Places in corolla tube	Adnate to corolla tube 3.0–3.6 mm from base	Adnate to corolla 3–11 mm above base	Adnate to corolla 4–10 mm above base	Adnate to corolla 1.8–4 mm above base	Adnate to corolla 1–2 mm above base
Free filaments length	3–7 mm	8–11 mm	6–10 mm	3.5–7 mm	ca. 4 mm
Staminode length	3.1–3.3 mm	ca. 0.5 mm	ca. 3 mm	ca. 1.5 mm	ca. 1 mm
Ovary	Glabrous	Glandular pubescent	Glabrous	Glabrous	Glabrous

### 3.2 Molecular Analyses

**FIGURE 6:**
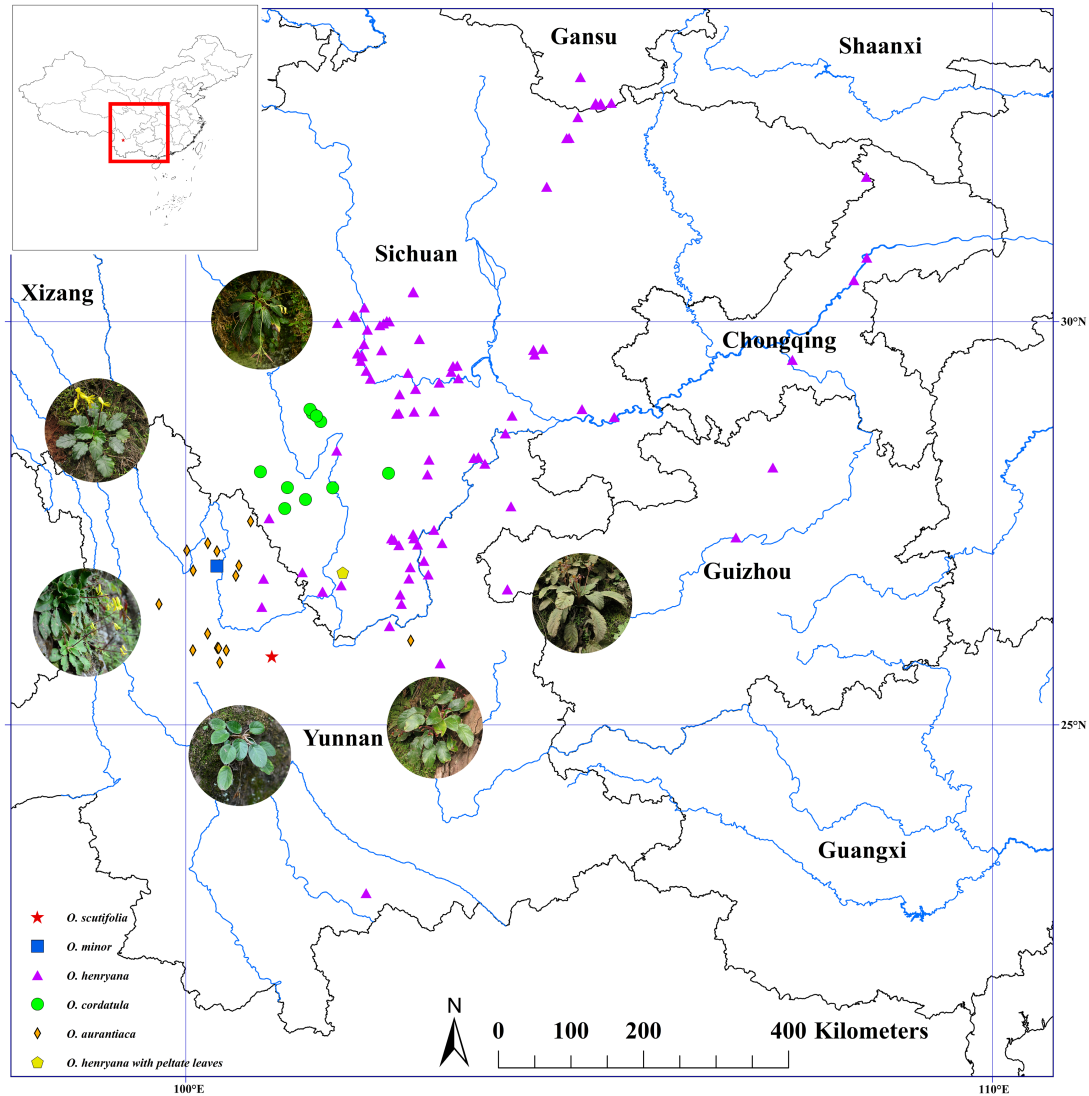
Distribution of *Oreocharis scutifolia*, *O.cordatula*, *O.aurantiaca*, *O.minor*, peltate and nonpeltate *O.henryana* based on our collections and specimens in herbaria. Photographs by Zhi Xie and Li‐Hua Yang.

A total of 112 taxa (including outgroups) were included in the molecular phylogenetic analysis (Figure 6), comprising 118 populations of *Oreocharis* (Table S1). The phylogenetic analyses strongly supported the clustering of the new species with *O. henryana*, *O. cordatula*, *O. minor* Pellegr., and *O. aurantiaca* (LPP = 1, Figure 6). Along with *O. cinnamomea* Anthony, these species form a clade with maximum support (LPP = 1, Figure 6). In addition, all of the six *O. henryana* populations (including the peltate‐leaved sample) formed a strongly supported clade (LPP = 1, Figure 5). The morphological evidence revealed its specificity, and molecular analysis confirmed its relationship with other *Oreocharis* species. As a result, the newly collected plant is described here as a new species, and named *Oreocharis scutifolia* Z. Xie & H. H. Kong sp. nov.

## 4 Discussion

The peltate leaves, a distinctive feature in *Oreocharis*, serve as an easily distinguishable characteristic for classifying new species based on living plants. However, the identification of one *O. henryana* population with peltate leaves introduces a layer of complexity to the taxonomic issue (Figure 3). It further suggests that the reliance solely on morphological evidence for describing new species is inadequate, and emerging developments in genomics may stimulate a more profound understanding of taxonomy (Karbstein et al. 2024).

Five nonpeltate *O. henryana* populations formed a strongly supported clade (LPP = 1, Figure 6), and the internal branch nodes also received strong support (LPP ≥ 0.92, Figure 6). The peltate *O. henryana* population formed a highly supported clade (LPP = 1, Figure 6) with the above five nonpeltate *O. henryana* populations and is situated at the base of this clade. It can be debated whether the peltate *O. henryana* population belongs to the same gene pool as the nonpeltate *O. henryana* populations, a hypothesis that necessitates further sampling for future research.

We thoroughly considered the ecological characteristics of the new species. It exclusively thrives in shady slopes on soils or, in some cases, on rocks, beneath *Quercus franchetii* Forests (Figure 1A,B), which is the climax plant community of the Dry–Hot Valley of the Jinsha River Basin in Dayao county, at an altitude of 2000–2200 m. The soil type in its habitat is classified as Dayao Red Purple Soil (Hu, Zhou, and Lv 2006). The Dry–Hot Valley of Jinsha River Basin experiences a rainy season from June to September each year, followed by a dry period that lasts from October through May of the ensuing year (Jin et al. 2000). These plants in this area complete their life cycle, including flowering and fruiting, during the brief four‐month span of the rainy season.

Meanwhile, the digitization of specimens is neither completed nor comprehensive across different herbaria. Therefore, it is impractical to examine all the specimens individually, and omissions are inevitable. Furthermore, when the new species are pressed into specimens, their floral features, including corolla shapes and colors, frequently become obscured, and the presence of peltate leaves may indistinct due to dehydration. The specimens of the newly described species, *O*. *cordatula*, *O*. *aurantiaca*, and even *O*. *henryana*, exhibit significant similarities, making it challenging to distinguish them even upon visual examination (Table 1). In the absence of physical specimens, differentiation becomes nearly impossible. Consequently, herbarium specimens identical to this new species could exist due to their similarity and difficulty in distinction but have not been observed during our examination.

### 4.1 Taxonomy Treatment

*Oreocharis scutifolia* Z. Xie & H. H. Kong sp. nov (Figure 1).

Type.

China, Yunnan Province, Chuxiong Yi Autonomous Prefecture, Dayao county, Shiyang community, Dashiqiao village, alt. 2,134 m, in the Dry–Hot Valley, rocks or soils on hillsides in the forests, flowering, July 12, 2023, Z. Xie & M. Zhang, XieZ 3677 (holotype, IBSC! isotype, IBK!).

### 4.2 Diagnosis

This species is morphologically distinct from other *Oreocharis* species, primarily due to its peltate leaves combined with yellow flowers.

### 4.3 Description

Herb perennial and stemless, rhizomatous. Leaves basal, arrangement spiral; peltate; petiole densely brown manicate lanose, 2.3–6.2 cm long; leaf blade leathery, oblong, ovate, oval to rounded, 2.8–7.7 × 1.8–4.5 cm long; lateral veins 5–7 on each side of midrib; adaxially green, smooth and glabrous, midrib veins depressed, lateral veins indistinct; abaxially brown, densely brown manicate lanose, midrib and lateral veins distinct; base rounded, apex obtuse, margin shallowly undulate to nearly entire. Inflorescence cymose, axillary, 2–6 per plant, 6–12 flowered per cyme. Peduncle 2–4 branched, 3–7 cm long, densely white glandular pubescent; bracts usually not present or caducous. Pedicel 1.2–1.8 cm long, densely white glandular pubescent. Calyx 2.4–3.0 mm long, 5‐sect nearly from base; segments equal, oblong, 1.9–2.7 × 1.1–1.3 mm, margin entire, apex obtuse, adaxially glabrous, abaxially sparsely white glandular pubescent. Corolla tubular, yellow, 1.1–1.3 cm long, outside white glandular pubescent and inside glabrous; tube cylindric, gradually narrowing toward mouth, 8–9 mm long, base of tube 2.9–3.2 mm in diam., middle of tube 1.8–1.9 mm in diam.; limb 2‐lipped, 3.6–3.9 mm long, 4.4–5.2 mm wide; adaxial lip 2‐lobed, 1.4–1.6 × 1.5–1.8 mm, apex obtuse; abaxial lip 3‐lobed, central lobe oblong, apex obtuse, 2.3–2.9 × 1.3–1.5 mm; lateral lobes ovate, apex rounded, 2.8–3.3 × 2.3–2.6 mm. Stamens 4, filaments glabrous; adaxial 6.1–6.6 mm long, adnate to corolla tube 3.0–3.4 mm from base; abaxial 3.3–3.6 mm long, adnate to corolla tube 0.8–1.1 mm from the base; anthers free, basifixed, 0.9–1.1 mm long, elliptic, 2‐loculed, dehiscing longitudinally; staminode 3.1–3.3 mm long, adnate to corolla tube 0.7–1.0 mm from base. Disk ring‐like, yellow, 1–2 mm high, margin shallowly 5‐lobed. Pistil glabrous, 0.5–1.6 cm long; ovary elliptic, 0.4–1.2 cm long, 1‐loculed; style 0.2–0.4 cm long; stigma 1, emarginate. Capsule straight, narrowly elliptic, glabrous, 2–3 × 0.3–0.5 cm. Seeds not seen.

### 4.4 Distribution, Habitat and Phenology

The new species is endemic to the Dry–Hot Valley of the Jinsha River Basin and is currently known to occur exclusively in Dashiqiao village, Shiyang community, Dayao county, Chuxiong Yi Autonomous Prefecture, Yunnan, China (Figure 4). It grows on rocks or in soil under semihumid evergreen broad‐leaved forests (Figure 1A). Flowering July to August, and fruiting August to September.

### 4.5 Conservation Status

To date, we discovered only one population of the new species in the field, located at the type locality on the hillside of the village, comprising ca. 500 mature individuals and covering ca. 10,000 m^2^ (100 × 100 m) area. This habitat is under severe threat from various human activities, including road construction, building, deforestation, and grazing. Furthermore, it has been continuously invaded by *Ageratina adenophora* (Spreng.) R. M. King & H. Rob. for an extended period, and this invasive species covers a substantial area while expanding within the vicinity, which directly jeopardizes the survival of the new species (Figure 1B). According to the guidelines to the IUCN Red List Categories and Criteria, the new species is hereby assessed as “Critically Endangered [CR, B1ab(iii), B2ab(iii)]” (IUCN Standards and Petitions Subcommittee, 2024). Therefore, the survival of this new species is in jeopardy, necessitating immediate protective measures.

### 4.6 Etymology

The species is named after its peltate leaves.

### 4.7 Vernacular Name

In Chinese mandarin “Dùn Yè Mǎ Líng Jù Tái” (盾叶马铃苣苔).

### 4.8 Key to the Similar Species With *O. scutifolia*

1, Leaf peltate. …………………………………………….…………………………2.

1, Leaf not peltate. …………………………………………………………………3.

2, Corolla purple to deep purple. ……*O. henryana*, only one population in Sichuan, China.

2, Corolla yellow. …………......…………………………………………....……*O. scutifolia*.

3, Pistil glabrous. …………………………………………………………………………..4.

3, Pistil covered with light brown glandular pubescence. ……………………..*O. cordatula*.

4, Corolla yellow. ………………………………………………………………………….5.

4, Corolla purple to deep purple. ………………*O. henryana*, all but one peltate population.

5, adaxially blade leaf glabrous. ………………………………………………… *O. minor*.

5, adaxially blade leaf puberulent. ……………………………………….....*O. aurantiaca*.

**Additional specimens of *O. scutifolia*.**

CHINA. Yunnan: Chuxiong Yi Autonomous Prefecture, Dayao county, Shiyang community, Dashiqiao village, alt. 2,077 m, in the Dry‐Hot Valley, rocks or soils on hillsides in the forests, flowering, August 4, 2024, Z. Xie, XieZ 4125 (IBSC).

## Author Contributions

**Zhi Xie:** formal analysis (lead), investigation (lead), methodology (lead), writing – original draft (lead). **Na‐Na Peng:** formal analysis (supporting), methodology (supporting), writing – review and editing (supporting). **Miao Zhang:** formal analysis (supporting), investigation (equal). **Guo‐En Ding:** investigation (supporting), visualization (supporting). **Fang Wen:** formal analysis (supporting), writing – review and editing (supporting). **Hang‐Hui Kong:** funding acquisition (lead), project administration (lead), writing – review and editing (lead).

## Conflicts of Interest

The authors declare no conflicts of interest.

## Supporting information


**Data S1.** List of sampled taxa and their GenBank accession numbers of transcriptome data. Species in bold were generated in this study, and the transcriptome data of other populations came from our previous study.

## Data Availability

The sequences of this study have been deposited in The National Center for Biotechnology Information (NCBI) database. The transcriptome data of *Oreocharis scutifolia*, *O. henryana* with peltate leaves, and *O. cordatula* in this study are openly available from NCBI: https://www.ncbi.nlm.nih.gov/sra/PRJNA1032259. GenBank accession numbers of transcriptome data in this study can be found in Table S1. The 574 matrices used for this study can be accessed at: 10.6084/m9.figshare.26927632.v1.
